# Analysis of the Gut Microbiome of Wild and Captive Père David’s Deer

**DOI:** 10.3389/fmicb.2019.02331

**Published:** 2019-10-04

**Authors:** Cheng-He Sun, Hong-Yi Liu, Bin Liu, Bao-Dong Yuan, Chang-Hu Lu

**Affiliations:** ^1^College of Biology and the Environment, Nanjing Forestry University, Nanjing, China; ^2^Jiangsu Dafeng Père David’s Deer National Nature Reserve, Yancheng, China; ^3^College of Biology and Food, Shangqiu Normal University, Shangqiu, China

**Keywords:** 16S rRNA, dietary factor, gut microbiome, Père David’s deer, population management

## Abstract

Père David’s deer (*Elaphurus davidianus* or milu) is a highly endangered species originating from China, and many deer are currently being raised in captivity for gradual re-introduction to the wild. Wild and captive deer currently live in the same region but have vastly different diets. In this study, we used 16S rRNA high-throughput sequencing to identify the healthy core microbiome in the gut of wild and captive Père David’s deer and investigate how dietary factors influence the gut microbiome by comparing their differences. A core shared gut microbiome was identified in healthy Père David’s deer, which was similar to that of other ruminants, mainly comprising the phyla Firmicutes and Bacteroidetes. There were no differences in the richness or diversity of the gut microbiome between the wild and captive deer. However, PCA and ANOSIM demonstrated clear differences in the microbial community structure between the captive and wild deer, which mainly manifested as changes in the relative abundance of 39 bacterial genera. As the majority of these genera were not dominant in the deer gut, no significant difference was detected in functional modules related to the microbiome between the two groups. Therefore, the difference in dietary factors does not appear to affect the healthy core gut microbiome between captive and wild Père David’s deer, suggesting strong co-evolution and the possibility of re-establishment in the wild. These data could guide future applications of population management in Père David’s deer conservation.

## Introduction

Père David’s deer (*Elaphurus davidianus*) originated in the middle and lower reaches of the Yangtze River but disappeared from China in the early twentieth century (Jiang et al., 2000). Fortunately, the surviving deer flourished in Britain. With the help of the World Wide Fund for Nature, some of the descendants of these deer were re-introduced to China in 1985 and fully adjusted to their native country (Jiang et al., 2000). Indeed, the population continued to develop, and there are currently thousands of Père David’s deer inhabiting more than 50 protected areas in China. This population has been growing so steadily that some of these deer have been returned to the wild for re-establishment of the wild population (Jiang et al., 2000; Yang et al., 2016). However, Père David’s deer remains a significant conservation concern in China. Mass die-offs of deer have occurred suddenly in some protected areas, which have been suggested to be related to infections of the gastrointestinal tract (Qiu et al., 2014; Bahrndorff et al., 2016; Yang et al., 2016; Zhang et al., 2018). Therefore, future conservation and management decisions require more focus and data on the health, welfare, and quality of Père David’s deer. In particular, studies on the metabolism, diet composition, and energy demand of Père David’s deer are needed.

The relation between the composition of the gut microbiome and health of the host animal is increasingly recognized and is of particular importance for endangered species that have experienced recent bottleneck events (Schwab et al., 2009). The gut microbiome forms a symbiotic relationship with the host as a result of their long-term coevolution (Shapira, 2016), in which the host provides a suitable habitat for gut microbes while the gut microbiome facilitates a variety of physiological activities for the host, such as gut immunity, metabolism, vitamin synthesis, and nutrient absorption (Andoh, 2016). The animal gut represents a complex ecosystem that is in a dynamic balance depending on the interplay of diet, host factors, and gut microbiome (Nagy-Szakal and Kellermayer, 2011). Numerous studies have indicated that these interactions between the gut microbiome and the host are very complex. For example, environment, diet, and disease can affect the microecological balance and health of the gut and thus impact the host as a whole (Waite et al., 2014; Kers et al., 2018).

Accordingly, dietary factors affect not only the condition of Père David’s deer but also its gut microbiome (Schwab et al., 2009). The diet of Père David’s deer varies depending on its living environment (Zhang et al., 2018), with stark differences in diet between wild and captive deer. Deer in captivity mainly consume processed food, whereas wild deer consume unprocessed plants native to the region. Along with the increase of the captive Père David’s deer population, more and more deer will be hopefully returned to the wild (Yang et al., 2016). Therefore, understanding the influence of this key difference between wild and captive Père David’s deer on the gut microbiome can contribute to assessing and ensuring the long-term viability of this species.

Here, we investigated the healthy gut microbiome of Père David’s deer and identified the core gut microbiome shared by this species using 16S rRNA high-throughput sequencing. We also analyzed how dietary factors might influence the gut microbiome by comparing the microbial structure and composition between captive and wild deer populations. These results can provide baseline data for continued efforts in Père David’s deer conservation, such as health assessments, disease treatment, and re-establishment of wild populations.

## Materials and Methods

### Study Site, Subjects, and Sample Collection

Fecal samples of the deer were obtained from Dafeng Nature Reserve (33°05′N, 120°49′E), located in East China on the shore of the Yellow Sea, which harbors the largest population of Père David’s deer (including wild and captive deer) in China. The reserve is divided into three core areas. The mudflat wetland is the third core area, which is covered by Gramineae, Cyperaceae, and Compositae plants that serve as the primary food sources for the wild deer in this area (Wu et al., 2011; Yuan et al., 2019). The captive populations are divided into two parts in the first and second core areas, respectively; the captive deer in this study were sampled from the second core area. In contrast to the wild deer habitat, there are barely any wild plants in this area due to the extensive activities of the large herd of deer. Therefore, the diet of the captive deer mainly consists of corn and alfalfa silage, along with some soymeal and bran. We collected a total of 13 stool samples in autumn, comprising six samples from wild deer and seven samples from captive deer. All of the deer in this area are adults, had not recently been provided antibiotics, and were confirmed to be in good health. Stool samples were collected immediately after excretion and stored in liquid nitrogen. To prevent soil contamination, only upper and middle layers of feces were selected for sampling, and some contaminated samples were excluded during data processing. After collection, the samples were sent to the laboratory and stored at −80°C until DNA was extracted.

### DNA Extraction, Amplification, and Sequencing

Total bacterial DNA was extracted using the QIAamp^®^ DNA Stool Mini Kit (QIAGEN, Germany). The quality and quantity of extracted DNA were checked using agarose gel electrophoresis and a NanoDrop ND-1000 spectrophotometer (Thermo Fisher Scientific, USA), respectively. Amplification of the V4-V5 region of the 16S rRNA gene was carried out with the universal primers 515F (5′-GTG CCA GCM GCC GCG GTA A-3′) and 907R (5′-CCG TCA ATT CMT TTR AGT TT-3′). To ensure accuracy of the amplification, Q5 High-Fidelity DNA Polymerase (New England Biolabs, USA) was used in hot-start polymerase chain reaction (PCR) under the following conditions: an initial denaturation at 98°C for 2 min, followed by 25 cycles of denaturation at 98°C for 15 s, annealing at 55°C for 30 s, and extension at 72°C for 30 s, with a final extension for 5 min at 72°C. PCR amplicons were analyzed by agarose gel electrophoresis. High-quality amplicons were purified with Agencourt AMPure beads (Beckman Coulter, USA) and then quantified with the PicoGreen dsDNA Assay Kit (Invitrogen, USA). After the individual quantification step, PCR amplicons were pooled in equal amounts, and pair-end 2 × 300-bp sequencing was performed using an Illumina MiSeq platform (Illumina, USA).

### Bioinformatics and Statistical Analyses

Paired-end reads were assigned to each sample based on unique barcodes. Quality filtering was performed according to the following criteria: sequence length < 150 bp and base-calling accuracy ≤99% (Chen and Jiang, 2014). FLASH v. 1.2.7 was used to merge the filtered reads overlapping by ≥10 bp, and then the valid sequences were generated (Magoč and Salzberg, 2011). Chimeric sequences were detected and discarded using the UCHIME algorithm within Mothur v. 1.31.2 (Edgar et al., 2011). The remaining sequences were identified as high-quality sequences and were clustered into operational taxonomic units (OTUs) at a 97% identity level using the UCLUST algorithm in QIIME v. 1.8.0 (Caporaso et al., 2010). To visualize the shared and exclusive OTUs among species groups, a Venn diagram was generated using R-based analysis of the occurrence of OTUs across groups regardless of their relative abundance (Zaura et al., 2009). Annotation of the OTUs was performed according to the SILVA rRNA database (Quast et al., 2013). OTUs accounting for less than 0.001% of total sequences across all samples were discarded. To minimize the difference of sequencing depths across samples, an average, rounded rarefied OTU table was generated by averaging 100 evenly resampled OTU subsets under the 90% of the minimum sequencing depth for further analysis (Liu et al., 2019; Sun et al., 2019).

QIIME v1.8.0 was used to calculate alpha diversity indices, including the Chao1, ACE, Shannon, and Simpson index (Caporaso et al., 2010). Principal components analysis (PCA) was performed based on the genus-level compositional profiles to reflect the difference of the microbial communities across samples using R packages v3.2.0 (Ramette, 2007). ANOSIM was carried out based on unweighted and weighted UniFrac distance to assess the difference of microbial community structures between the wild and captive deer (Warton et al., 2012). Metastats was carried out to identify the critical bacteria causing the observed differences between the wild and captive deer at the phylum and genus levels (White et al., 2009; Segata et al., 2011). To account for false positives arising from statistical comparisons, we applied an FDR analysis to calculate Q-values (Bolnick et al., 2014). Kyoto Encyclopedia of Genes and Genomes (KEGG) pathway analysis was performed in PICRUSt to predict the biological functions of the identified taxa in the deer gut microbiome (Langille et al., 2013). Differences in the gut microbiome between the wild and captive deer were statistically evaluated through independent-samples t-test in IBM SPSS Statistics 19 (Field, 2005).

## Results

### Metadata and Sequencing

**Figure 1:**
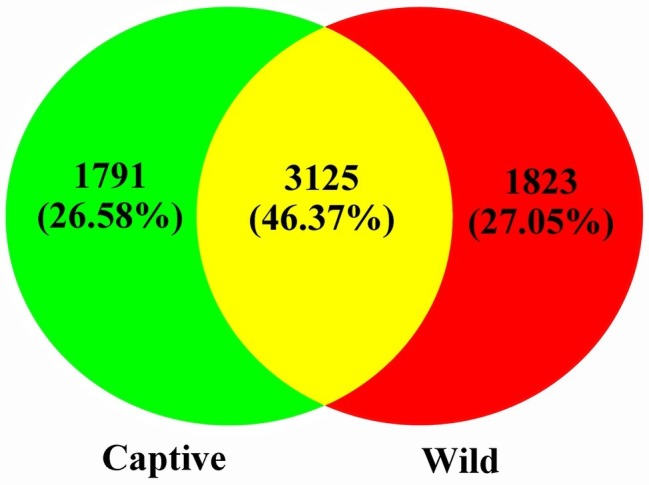
Venn diagram showing the number of shared OTUs among the captive and wild deer.

**Figure 2:**
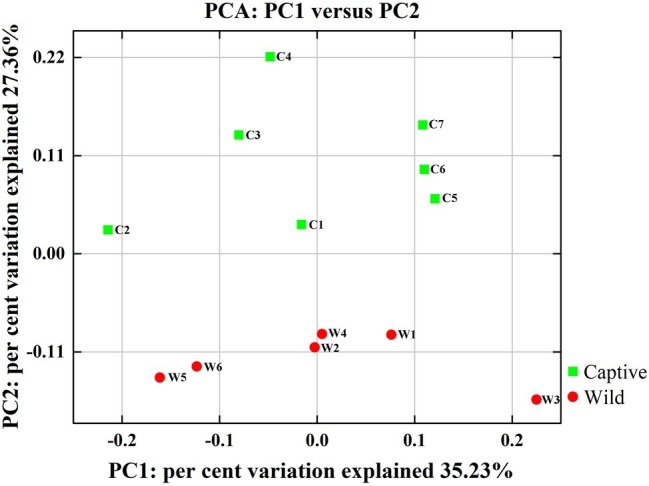
Two-dimensional principal components analysis score plot of the gut microbiome in all samples at the genus level based on Euclidean distance method.

A total of 551,963 valid sequences were obtained from the 13 stool samples (Table 1). After deletion of chimeric sequences, 368,004 high-quality sequences were retained (Table 1). These sequences clustered into 58,278 OTUs with 97% similarity. Through normalized processing, a total of 6,739 OTUs were retained for all further downstream analyses. The average number of OTUs before and after captive and wild population normalization was 10069.57 ± 3011.31 (mean ± SD); 7972.67 ± 994.41; and 2004.29 ± 136.62; 2017.50 ± 214.85, respectively. In the gut of Père David’s deer, the actual number of OTUs was 4,916 and 4,918 for captive and wild deer, respectively. As shown in Figures 1, 2, 125 OTUs (46.37%) were shared among captive and wild deer. Moreover, 3,614 OTUs (53.63%) were detected in only one population (Figure 1).

**Table 1:** Statistics of valid sequences and high-quality sequences.

Sample	Number of samples	Valid sequences		High-quality sequences	
		Total	Mean ± SD	Total	Mean ± SD
Captive deer	7	342,532	48,933 ± 18,038	232,750	33,250 ± 13,014
Wild deer	6	209,431	34,905 ± 5,613	135,254	22,542 ± 3,164
All deer	13	551,963	42,459 ± 15,126	368,004	28,308 ± 10,942

### Potential Core Gut Microbiome of Père David’s Deer

**Figure 3:**
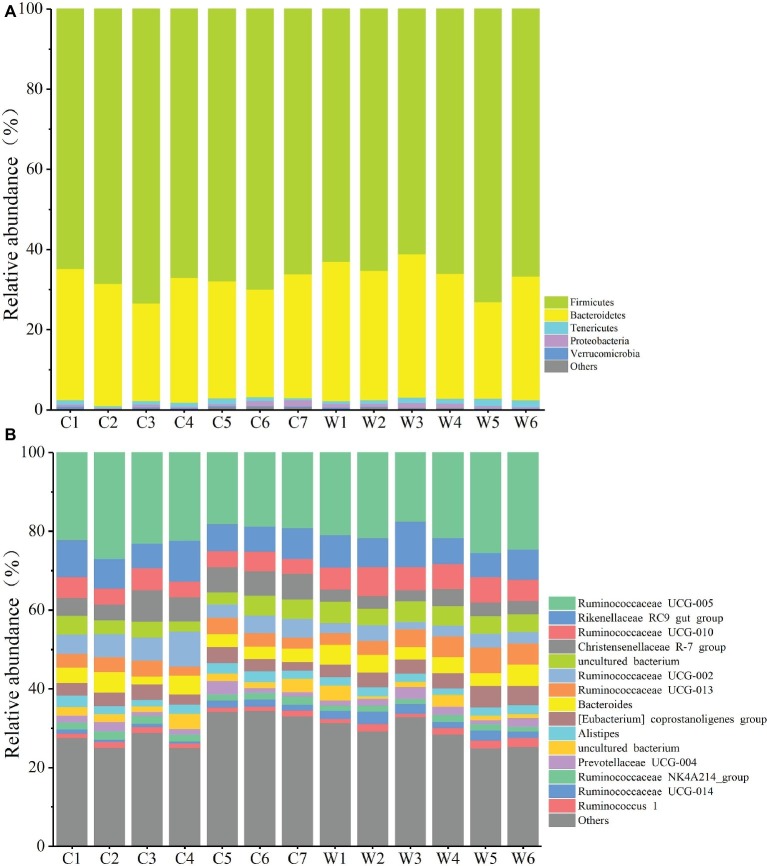
Relative abundance of different OTUs at the phylum **(A)** and genus **(B)** level for each sample.

To define the core microbiome in the gut of Père David’s deer, the number and relative abundance of shared OTUs at the genus level were calculated. Each captive deer shared 106 OTUs, which accounted for 51.20% of the total OTUs in captive deer. These OTUs contributed 98.71 ± 4.4% to the total abundance of the gut microbiome (Table 2). The wild deer shared 99 OTUs accounting for 48.53% of the total OTUs, which contributed 97.44 ± 1.05% to the total abundance (Table 2). All 13 individuals shared 88 OTUs, accounting for 36.51% of the total OTUs in both captive and wild deer, accounting for 96.61 ± 0.89% of the total abundance per individual (Table 2). The shared OTUs mainly comprised of bacterial genera in the phyla Firmicutes and Bacteroidetes (Table 3, Figure 3). The most important contributor was the genus Ruminococcaceae UCG-005 in the phylum Firmicutes with an average relative abundance of 21.65 ± 2.87% per deer (Table 3, Figure 3). The nine other highest contributors were Rikenellaceae RC9 gut group, Ruminococcaceae UCG-010, Christensenellaceae R-7 group, uncultured bacterium, Ruminococcaceae UCG-002, Ruminococcaceae UCG-013, *Bacteroides, [Eubacterium] coprostanoligenes* group, and *Alistipes* (Table 3, Figure 3).

**Table 2:** Number and relative abundance of shared operational taxonomic units (OTUs) at the genus levels in Père David’s deer.

Sample	Number of samples	Number of total OTUs	Number of shared OTUs	Relative abundance of shared OTUs (mean ± SD)
Captive deer	7	207	106	98.71 ± 4.4%
Wild deer	6	204	99	97.44 ± 1.05%
All deer	13	240	88	96.61 ± 0.89%

**Table 3:** Relative abundance of the 10 most abundant bacteria in the gut of Père David’s deer.

Phylum	Genus	Relative abundance (Mean ± SD)		
		Captive deer	Wild deer	All deer
Firmicutes	Ruminococcaceae UCG-005	21.44 ± 3.11%	21.88 ± 2.83%	21.65 ± 2.87%
Bacteroidetes	Rikenellaceae RC9 gut group	7.76 ± 1.54%	7.92 ± 1.91%	7.84 ± 1.65%
Firmicutes	Ruminococcaceae UCG-010	4.54 ± 0.73%	6.15 ± 0.68%	5.29 ± 1.08%
Firmicutes	Christensenellaceae R-7 group	5.96 ± 1.32%	3.37 ± 0.51%	4.77 ± 1.67%
Bacteroidetes	Uncultured bacterium	4.00 ± 0.93%	4.79 ± 0.51%	4.36 ± 0.84%
Firmicutes	Ruminococcaceae UCG-002	5.43 ± 1.73%	2.93 ± 0.71%	4.27 ± 1.84%
Firmicutes	Ruminococcaceae UCG-013	3.45 ± 0.69%	4.72 ± 1.29%	4.03 ± 1.16%
Bacteroidetes	*Bacteroides*	3.67 ± 1.08%	4.24 ± 0.88%	3.93 ± 1.00%
Firmicutes	*[Eubacterium] coprostanoligenes* group	3.22 ± 0.71%	4.12 ± 0.89%	3.64 ± 0.90%
Bacteroidetes	*Alistipes*	2.34 ± 0.44%	2.07 ± 0.24%	2.21 ± 0.38%

### Difference in Gut Microbial Communities

**Figure 4:**
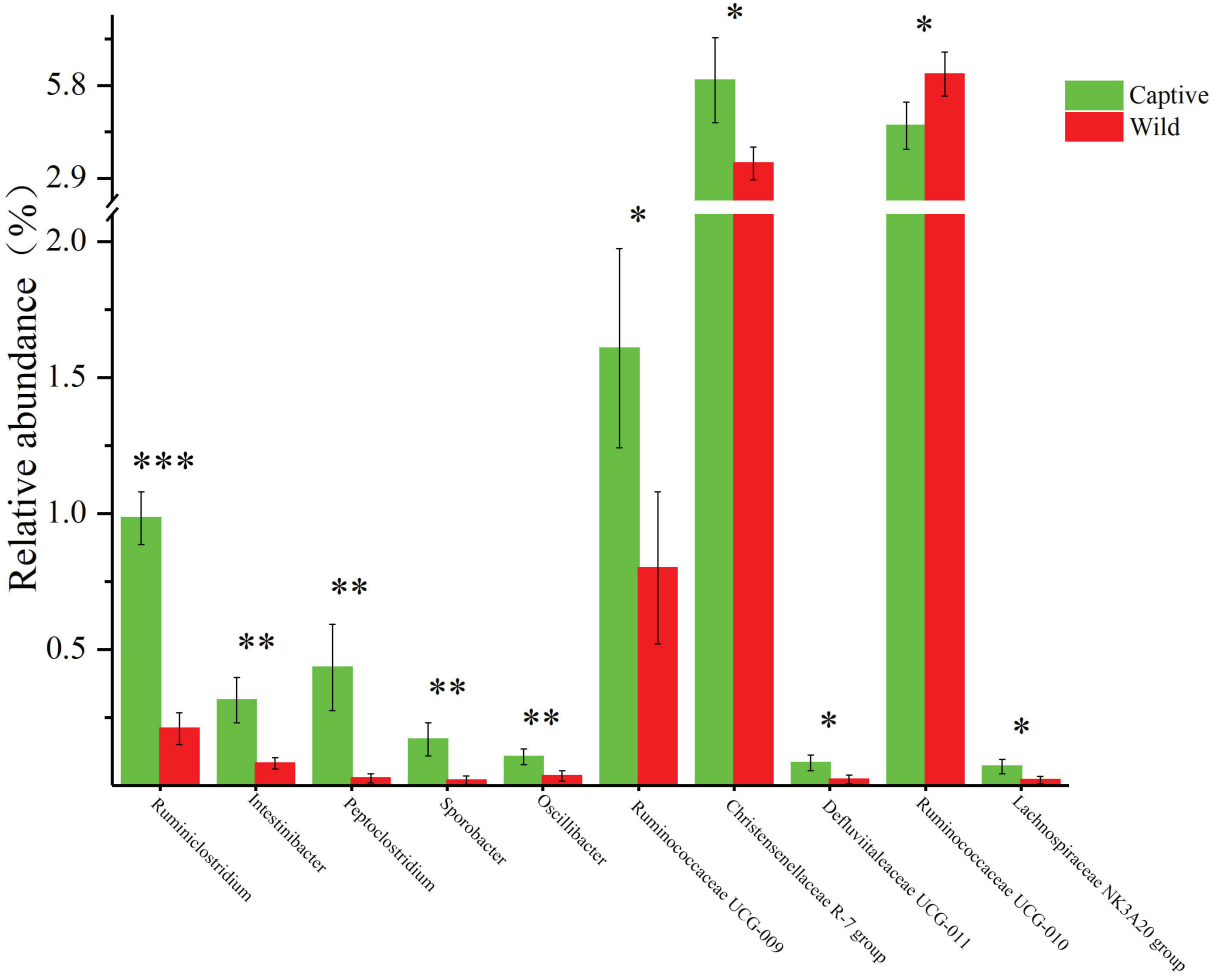
The 10 most variable bacterial genera between captive and wild Père David’s deer. ^*^Q < 0.05, ^**^Q < 0.01, ^***^Q < 0.001.

All alpha diversity indices of the gut microbial communities in captive deer were marginally higher than those of wild deer, but the differences were not significant (Table 4). This indicated no visible change in the richness and diversity of gut microbial communities between captive and wild deer. However, the PCA result showed more similarity of the microbial community structure within each deer population (Figure 2). ANOSIM, which is based on the Unifrac distance, further confirmed the significant difference in the microbial community structure between the captive and wild deer (*R* = 0.8466, *p* = 0.001 for unweighted Unifrac; *R* = 0.5317, *p* = 0.002 for weighted Unifrac). Metastats was used to identify the key bacteria responsible for the difference between wild and captive deer, revealing substantial differences in the relative abundances of 39 bacterial genera, including *Ruminiclostridium*, *Intestinibacter*, *Peptoclostridium*, *Sporobacter*, *Oscillibacter*, Ruminococcaceae UCG-009, Christensenellaceae R-7 group, Defluviitaleaceae UCG-011, Ruminococcaceae UCG-010, and Lachnospiraceae NK3A20 group as the top 10 most variable bacterial genera (*p* < 0.001 and *Q* < 0.05) between populations (Figure 4). Except for Ruminococcaceae UCG-010, the relative abundances of the other nine bacterial genera were significantly higher in captive deer than in wild deer (Figure 4).

**Table 4:** Comparison of alpha diversity indices between captive and wild Père David’s deer.

Index	Captive deer (mean ± SD)	Wild deer (mean ± SD)	*p* (*t*-test)
Chao 1	2347.986 ± 345.960	2287.675 ± 613.454	0.837
ACE	2480.550 ± 430.915	2315.827 ± 653.557	0.597
Shannon	9.320 ± 0.189	9.405 ± 0.129	0.372
Simpson	0.994 ± 0.002	0.994 ± 0.001	0.499

### Functional Modules of Gut Microbial Communities

**Figure 5:**
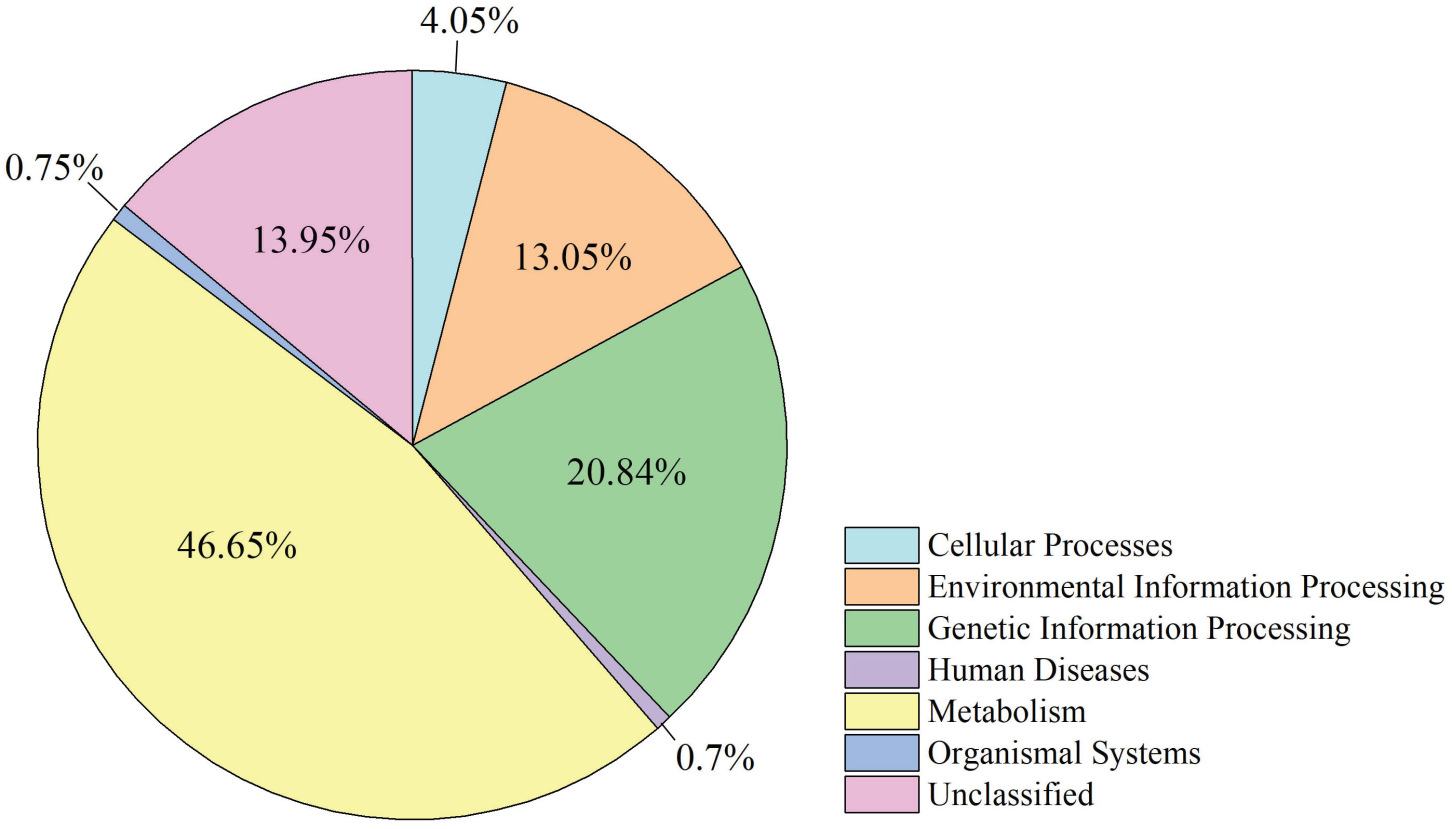
Distribution of functional OTUs defined by KEGG pathways for all samples.

**Figure 6:**
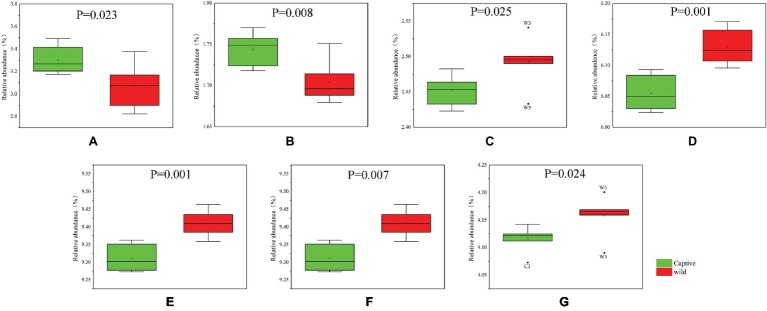
Functional OTUs responsible for the difference of KEGG pathways between captive and wild Père David’s deer: **(A)** cell motility; **(B)** signal transduction; **(C)** folding, sorting, and degradation; **(D)** translation; **(E)** replication and repair; **(F)** metabolic diseases; **(G)** nucleotide metabolism.

The functional modules related to the deer gut microbiome were predicted using the KEGG database. Overall, functions could be assigned to 86.05% of the OTUs, while the functions of 13.95% of the OTUs were unknown (Figure 5). At the first level of KEGG pathway analysis, there was no significant difference in the functional OTUs between captive and wild deer. The OTUs involved in metabolism were the most abundant, which accounted for 46.65% of all functionally annotated OTUs, followed by those involved in genetic information processing and environmental information processing (Figure 5). However, at the second level, some differences in the makeup of functional OTUs were observed between captive and wild deer (Figure 6). In particular, the abundance of OTUs involved in cell motility and signal transduction were higher in captive deer, whereas there were more OTUs involved in folding, sorting and degradation, translation, replication and repair, metabolic diseases, and nucleotide metabolism in wild deer (Figure 6).

## Discussion

As an important and rare wild animal in China, the Père David’s deer plays a key role in demonstrating that other endangered species can be protected through the *ex situ* conservation of existing deer populations as well as the application of artificial management and reproduction to expand their populations (Zhang et al., 2018). Since the release of captive Père David’s deer in Yancheng Reserve, the number of wild elk has been increasing. However, owing to technical limitations, there is relatively limited comparative analysis of the health status of captive and wild Père David’s deer; especially, the analysis of the differences in their gut microbiota is lacking. In this study, the core microbiome of captive and wild Père David’s deer was compared, and the diversity of microbial communities was analyzed.

The gut microbiota in the captive and wild Père David’s deer showed a high degree of similarity (97.43%) (Figure 3A), with Firmicutes and Bacteroidetes being the two most common phyla. This finding is similar to that in other ruminant animals (Kim et al., 2014; Tanca et al., 2017; Zhang et al., 2018). The dominant genera (Table 3, Figure 3B) identified in this study were similar to those reported by Wang et al. (2019); however, they only analyzed the microbiome up to the family level, and therefore a detailed comparison between our and their results cannot be made. Although Zhang et al. (2018) reported 10 dominant genera in Père David’s populations in Beijing and Shishou, only three of those genera were identical to the 10 dominant genera found in this study. The difference of seven dominant genera may be due to differences in the geographical location and climatic conditions of the sampling sites. Based on the analysis presented in Figure 2, we observed that there were greater differences between the captive and wild Père David’s deer populations, while the differences in microbial communities within the same populations were smaller.

Despite having different diets and exposure to some distinct environmental factors, we still found a potential core gut microbiome that is shared by both wild and captive Père David’s deer (Table 3, Figure 3). This core gut microbiome might be essential to health and function so that it has been retained even in the face of environmental changes. Père David’s deer as the host and its core gut microbiome could select each other to form a superorganism after long-term co-evolution (Shapira, 2016). This core gut microbiome might be transmitted vertically over generations, playing essential roles in many physiological activities of Père David’s deer (Asnicar et al., 2017). In particular, we found a high abundance of the family Ruminococcaceae in the gut of Père David’s deer. The members of this family are well-established cellulose utilizers (Biddle et al., 2013). Moreover, the core gut microbiome in Père David’s deer is similar to that reported in cattle, sheep, and other ruminants, indicating their close genetic relationship and similar diet (Shanks et al., 2011; Kim et al., 2014; Tanca et al., 2017).

As seen in Table 2, although the different taxa found in captive and wild Père David’s deer are rare (only 4% of the total microbial composition), they constitute 63% of a unique community. This may be due to the differences in the diets of captive and wild Père David’s deer. In the wild, Père David’s deer mainly consume unprocessed plants, including those of the families Gramineae, Cyperaceae, and Compositae. By contrast, captive Père David’s deer feed mainly on corn and alfalfa silage, along with a high-protein diet of soymeal and bran. The influence of dietary factors on host gut microbial communities has been widely demonstrated (Nelson et al., 2013; Wetzels et al., 2015; Li et al., 2017). Therefore, the differences in the gut microbial communities observed between the captive and wild Père David’s deer in this study likely reflect these dietary differences (Figure 2). Nevertheless, the 39 bacterial genera contributing to this separation were not the dominant genera in the gut of Père David’s deer (Figure 4). Since the deer sampled in this study were all healthy, these 39 bacterial genera that showed the greatest amount of change might have a relatively negligible impact on the physiological activities of Père David’s deer. Although the captive deer ate mainly silage, the relative abundance of *Lactobacillus* and *Bifidobacterium* did not increase with respect to that of wild deer. There was also no evidence that silage could enhance the richness and diversity of the gut microbiome (Table 4). Therefore, our findings indicate that the healthy core gut microbiome of Père David’s deer might not be largely influenced by dietary factors, but is rather maintained at an extremely stable state for proper function.

Undoubtedly, we were mainly interested in determining the biological functions of the gut microbiome. Given the finding of a core gut microbiome, there was no significant difference in the makeup of functional OTUs at the first level of KEGG pathway analysis between captive and wild deer (Figure 5). The gut microbiome in Père David’s deer was found to be mainly related to metabolism, confirming a primary role in aiding host digestion and absorption (Dai et al., 2011). Moreover, a high proportion of OTUs was found to be involved in environmental information processing, suggesting an essential role of the gut microbiome in facilitating adaptation to changing environments (Shapira, 2016). However, more specific functions of the gut microbiome in Père David’s deer could not be revealed due to the limitation of 16S rRNA sequence analysis and annotation. Nevertheless, some differences in the composition of functional OTUs were found at the second level of KEGG analysis between captive and wild Père David’s deer (Figure 6), which further highlights the need for future metagenomic investigations on the gut microbiome of this endangered population.

In conclusion, we identified the healthy core microbiome from the gut of wild and captive Père David’s deer and investigated how dietary factors influence the gut microbiome by comparing them. Differences in diet do not appear to affect the healthy core gut microbiome of captive and wild Père David’s deer, suggesting strong co-evolution and the possibility of re-establishment of the microbiota in the wild deer. Our data expand the understanding of the health status of wild Père David’s deer populations and provide a scientific basis for monitoring the health status of the Père David’s deer.

## Data Availability Statement

DNA sequences and metadata file have been uploaded to Figshare available at https://doi.org/10.6084/m9.figshare.8039915

## Ethics Statement

The animal study was reviewed and approved by this research was approved by the Ethics Committee of the Nanjing Forestry University and the Jiangsu Dafeng Père David’s Deer National Nature Reserve. This study did not involve any animal tissues. All fecal samples were collected by the keepers to avoid stress reaction of these deers.

## Author Contributions

H-YL and C-HL contributed to the experimental design. BL and B-DY were involved in the sample collection and pre-processing. C-HS contributed to data analysis and image editing. H-YL and C-HS drafted the manuscript. C-HL reviewed and edited the manuscript.

### Conflict of Interest

The authors declare that the research was conducted in the absence of any commercial or financial relationships that could be construed as a potential conflict of interest.
